# Comparative analysis between different volumetric methods on measuring intracranial hemorrhage incorporating roundness index

**DOI:** 10.1371/journal.pone.0292092

**Published:** 2023-10-03

**Authors:** Supanut Chaidee, Papangkorn Inkeaw, Thampaphon Makee, Kamoltip Khamyod, Salita Angkurawaranon, Patrinee Traisathit, Tanat Vaniyapong, Imjai Chitapanarux

**Affiliations:** 1 Department of Mathematics, Faculty of Science, Chiang Mai University, Chiang Mai, Thailand; 2 Data Science Research Center, Department of Computer Science, Faculty of Science, Chiang Mai University, Chiang Mai, Thailand; 3 Global Health and Chronic Conditions Research Center, Chiang Mai University, Chiang Mai, Thailand; 4 Department of Radiology, Maharaj Nakorn Chiang Mai Hospital, Faculty of Medicine, Chiang Mai University, Chiang Mai, Thailand; 5 Data Science Research Center, Department of Statistics, Faculty of Science, Chiang Mai University, Chiang Mai, Thailand; 6 Neurosurgery Division, Department of Surgery, Faculty of Medicine, Chiang Mai University, Chiang Mai, Thailand; University of Pisa, ITALY

## Abstract

Intracranial hematoma (ICH) volume is considered a predictor of clinical outcome and mortality rate in ICH patients with traumatic brain injury (TBI). The ABC/2 method for ICH volume is the standard method used to date, however, its level of accuracy has been questioned in some studies. This study compared the performance of the ABC/2 method with planimetry and truncated pyramidal methods to highlight the potential of the planimetry method applied with automatic segmentation for evaluation of epidural hematoma (EDH) and intraparenchymal hematoma (IPH) volume. Six different phantoms were designed to evaluate the accuracy of volume estimation methods. 221 hematoma regions extracted from CT scans of 125 patients with head injury were also used to analyze the efficiency. The roundness index was utilized for the quantification of the ellipsoid-like shape. Regions of EDH and IPH on the CT scans were annotated by radiologists. The estimation errors for each method were statistically analyzed and compared. In addition, the relationship between the errors and roundness index was examined. The planimetry method showed the lowest relative error on phantom data. In the case of the CT scan data, the truncated pyramidal method resulted in the underestimation of the volumes of EDH and IPH. Meanwhile, the ABC/2, through principal component analysis (PCA) in the two-dimensional and PCA in the three-dimensional methods, resulted in a significant overestimation. In addition, both these approaches produced relative errors that showed a correlation with the roundness indexes for IPH. In comparison to other methods, the planimetry method had the lowest level of error with regards to calculation of the volume and it was also independent of the hematoma shape. The planimetry method, therefore, has the potential to serve as a useful tool for the assessment of ICH volume in TBI patients by using a deep learning system.

## Introduction

Traumatic brain injury (TBI) is a worldwide threat, having the highest incidence in the younger population demographic [1] and constitutes the major cause of death and severe disability [2]. The critical determinant of patient outcome is early recognition and timely, appropriate treatment of TBI, Computer Tomography (CT) being the gold standard technique for brain injury evaluation, playing a critical role in decision-making for surgery [3].

The intracranial hematoma (ICH) volume is considered to be the main predictor of clinical outcomes and mortality for ICH patients [4–8]. The volumes of epidural hematoma (EDH) and intraparenchymal hematoma (IPH) are indicators in decision making for surgical treatment of TBI patients [2]. Rapid and accurate measurement of hematoma volume is essential for clinical management. The rough approximation of the volume using the ABC/2 model has been widely used in the past decades for volume measurement and has been validated in studies into ICH. This method is derived from an approximation calculated in accordance with the formula for ellipsoids with axes A, B and C [6, 9–12]. Recently, many studies have presented machine learning models for hematoma segmentation which can identify the boundaries of hematoma regions [13–15]. Estimating hematoma volumes from the segmentation results are faster and more convenient. However, those studies focused on only the segmentation step. Some studies [13, 14] did not perform volume quantification while a study [15] lacks the detail of the volume approximation method used. Currently, there are a variety of methods for measuring hematoma [8] including planimetry volume calculation and truncated pyramidal method. The planimetry method is a simple method for approximating the hematoma volume in each CT slice as a disk, but it requires efficient computational models and appropriate extensive physician experience to calculate an accurate volume. Another method is the truncated pyramidal method, the use of volume approximation based on the similarity between two sheets, as presented in one study [16].

The volume estimation using ABC/2 is based on the assumption that the shape is a relatively regular ellipsoid. The concept of the roundness index is studied to determine the roundness of the shape of object. This is particularly useful in the field of geology [17–19]. The roundness index is a measure of how round a shape and can be used to determine how close a shape is to a regular ellipsoid. Application of this concept can be used to clarify the relationship between the roundness index and the relative error when calculating the volume of the shape. However, there is limited research into using automated systems to assess the roundness index. Studies assessing the accuracy of ABC/2 in association with a large number of traumatic hemorrhages and the clinical significance of accurate volumetric lesions in TBI have yet to be completed [3].

This study aims to compare the performance of the ABC/2 method to that of the computer-assisted planimetric technique and truncated pyramidal methods for the estimation of the volume of ICH and to identify the correlative factors that affect the accuracy of the ABC/2 method. The study will also include an investigation into the relationship between the roundness index and relative error using an automated computation system. The effects of ICH volumes computed by the computational method and radiologists on the surgical treatments are also analyzed.

## Materials and methods

### Datasets

#### Phantoms

**Fig 1:**
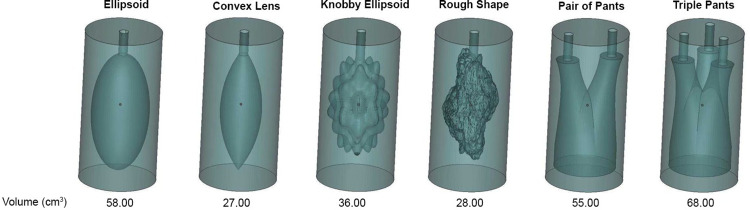
The six shapes of phantoms used in the experiments with the exact volumes.

To understand the volume approximation methods six phantoms representing different possible topological structure of shapes, as shown by Boissonnat, J [20], were designed and constructed using a 3D printer and then filled with water. The exact volumes of the phantoms were known for the purpose of analyzing the accuracy of volume assessment methods as illustrated in Fig 1. CT data of the phantoms were acquired using a CT scan (SOMATOM definition, Siemens, Germany) with 1.5 mm slide thickness and stored as a 512×512 pixel DICOM image. Voxels with an HU of 5–8 were determined as regions of water in the images.

#### Real dataset

Following an institutional review board-approved protocol, head CT scans of 162 patients with head injury/trauma were used to analyze the efficiency of volume assessment methods. The CT scans were retrospectively collected from adult patients (≥15 years old) during emergency department visits at Maharaj Nakorn Chiang Mai Hospital from January 1, 2014, to December 31, 2014. Inclusion criteria included patients who met the following conditions: head injury, head trauma, fall and traumatic intracranial hemorrhage. Exclusion criteria included: (1) Follow-up CT studies of patients with known recent TBI; (2) Studies of patients with recent neurosurgical intervention; (3) Patents without EDH and IPH; (4) Severe artifacts that degraded study quality i.e., motion artifact and metallic artifact; (5) No medical report found. We used only an initial non-contrast head CT scan of each patient in our analysis. The CT scans were acquired using two different machines at the institute (Toshiba Aquilion 16 or Siemens SOMATOM Definition). Each slide was a 512 × 512 pixel DICOM image. A CT scan may consist of more than one region of different hemorrhage types. Only regions of EDH and IPH on CT scans were analyzed. They were annotated by radiology residents and medical students who were trained by a board-certificated diagnostic neuroradiologist using the Fujifilm Synapse 3D. All annotated data were then refined and validated by a neuroradiologist. Each hemorrhage region was separately computed and analyzed. The regions whose volume was less than 0.5 cm^3^ were excluded. In total, we accrued 221 pieces of data pertinent to the hemorrhage regions including 105 EDHs and 116 IPHs from 125 patients in our analysis.

**Fig 2:**
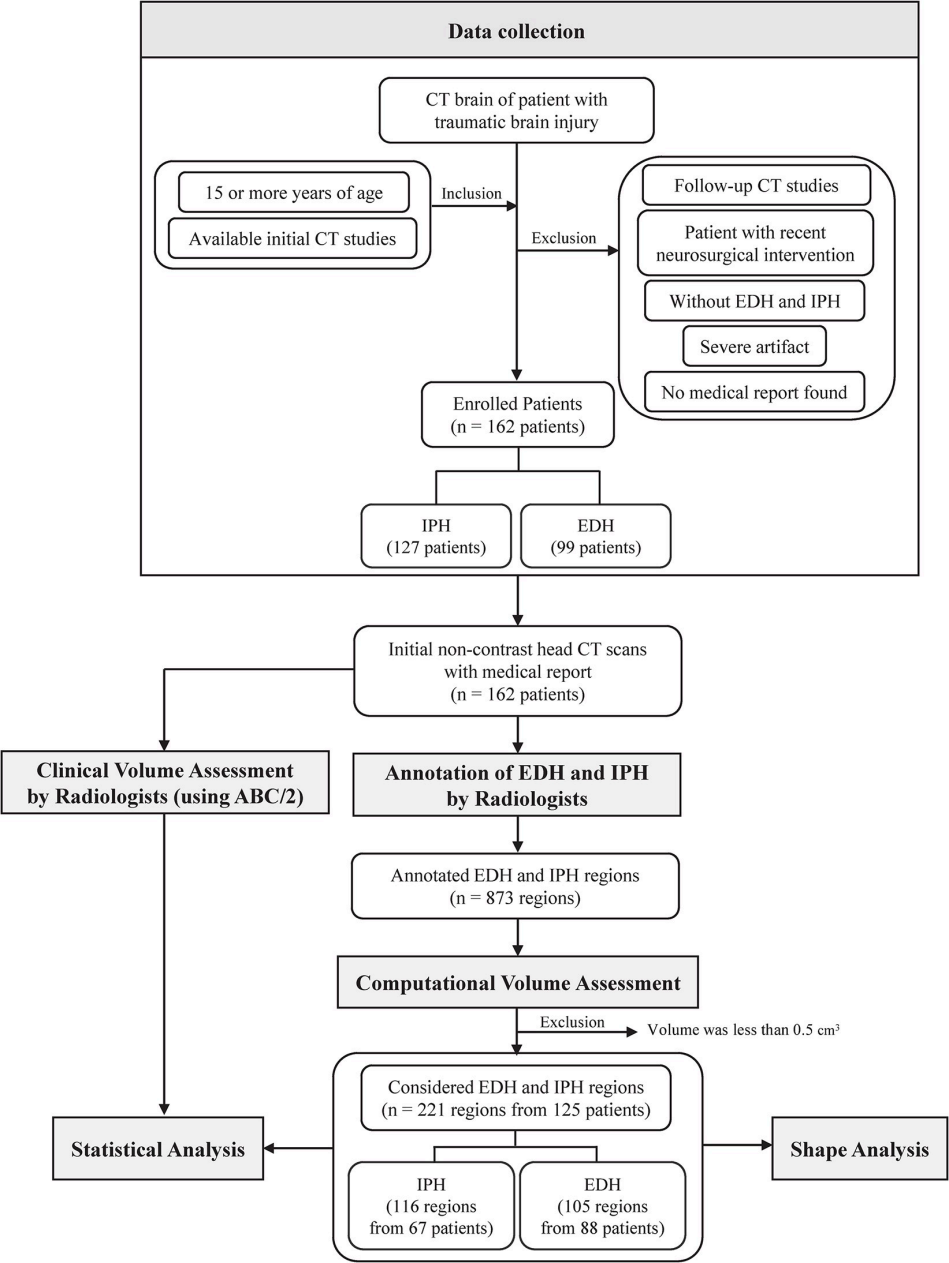
Flowchart of the methodology performed in this study.

One of the variables used to determine the need for surgical intervention is the size or volume of the hematoma. However, numerous criteria influence the decision of the neurosurgeon in opting to perform surgery including the location of the hematoma, coagulopathies, and the patient’s age, neurologic condition, cerebral perfusion pressure, hydrocephalus, and intracranial hypertension [21]. Patients with Glasgow Coma Scale (GCS) scores of 6 to 8 with frontal or temporal contusions exceeding 20 cm^3^ in volume with midline displacement of at least 5 mm and/or cisternal compression on CT scan, and patients with any lesion exceeding 50 cm^3^ in volume, are recommended to undergo surgery. Regardless of the patient’s Glasgow Coma Scale (GCS) score, a surgical evacuation is required for an epidural hematoma (EDH) larger than 30 cm^3^. An EDH less than 30 cm^3^ and with less than a 15-mm thickness and with less than a 5-mm midline shift in patients with a GCS score greater than 8 without focal deficit can be managed nonoperatively, with serial CT scanning and close neurological observation in a neurosurgical center [2]. The need for surgical intervention for each patient was queried from medical records to be used in our analysis. A flowchart of the methodology of this study is available in Fig 2.

We confirm that this study was approved and monitored by the Ethics Committee of the Faculty of Medicine, Chiang Mai University, Thailand (No. EXEMPTION 7870/2021). All methods were performed in accordance with the relevant guidelines and regulations. All head CT scans were fully anonymized before we accessed them. Due to the nature of the study, the ethics committee waived the requirement for informed consent.

### Computational volume assessment methods

**Fig 3:**
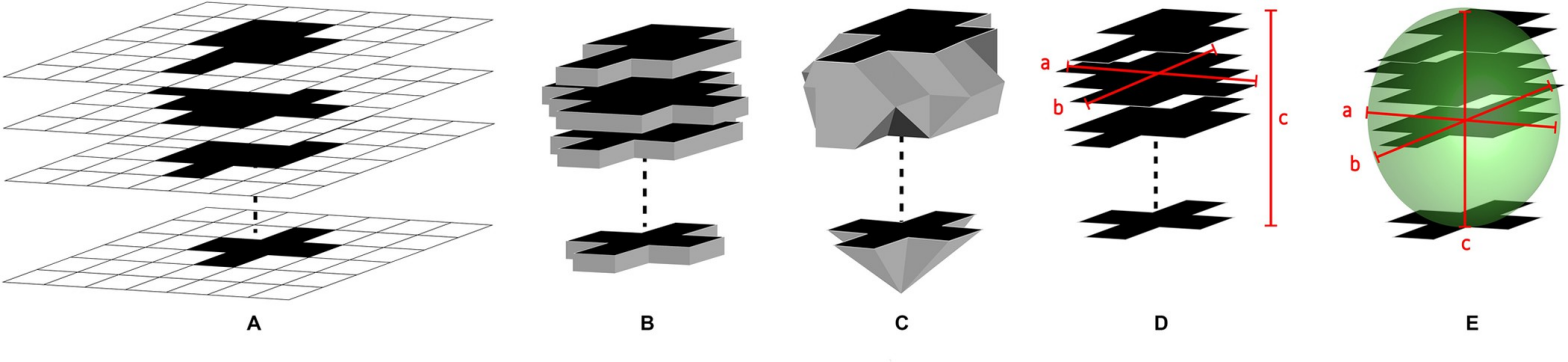
The visualization of four volume assessment methods. (A) Image to show the sequence of CT images; (B–E) the interpretation of planimetry, truncated pyramidal, ABC/2 through PCA in two-dimensional, and PCA in three-dimensional methods, respectively.

We assumed that a sequence (*X*_*1*_,*…*,*X*_*n*_) of the images is given so that each image keeps the same distance *ΔX*. Each image is represented by a binary digit (0 and 1) that identifies the region selected by a radiologist for volume computation, i.e., 1 if the region is selected and 0 if not. Four methods of volume assessment were investigated: planimetry, truncated pyramidal, ABC/2 through principal component analysis (PCA) in two-dimensional and PCA in three-dimensional methods. The characteristics of each method are shown in Fig 3. It is noteworthy that the truncated method is not significantly different from the disk method, however, we have mentioned it in this study to enable comparison with an earlier study by Sun and Sun. [16].

### Planimetry method

This method is used to approximate the volume of the three-dimensional shape of the real 3D shape based on the discrete information of voxels. For each sheet *X*_*i*_, the number of pixels that are set to *N*_*i*_ is counted, which represents the area of the shape on that sheet. The volume *V*_*i*_ of the shape on that sheet is then calculated by multiplying the area by the side length *s* of a square pixel with *s*^*2*^ and by the thickness of the sheet *ΔX*. In brief, the volume can be interpreted as the product of the number of voxels in the segmentation and the volume of a single voxel. That is, the total volume of the shape that appears in the sequence (*X*_*1*_,*…*,*X*_*n*_) is rigorously calculated using

$$V=\sum_{i=1}^{n}V_{i}=\sum_{i=1}^{n}N_{i}s^{2}\Delta X.$$

### Truncated pyramidal method

If a region A with the area *A*_*i*_ of a sheet *X*_*i*_ is connected to a region B with the area *B*_*i*_ of the sheet *X*_*i+1*_, we expect that the boundary of the region A and B would connect as a part of the lateral side of a truncated pyramid. Although the lateral side can be approximated using techniques described by Boissonnat, J. [20] and summarized in the study by Sun and Sun [16], we assume that the volume can be approximated using the truncated pyramid, in which the base of the pyramid is the region A or B. The volume *V*_*k*_ of the region bounded by the region A and B is approximated using

$$V_{k}=\frac{1}{3}(A_{i}+B_{i}+\sqrt{A_{i}B_{i}})\Delta X.$$

If the shaded region exists on the first and last sheets, we infer that the area of the prior and subsequent sheets is 0. The total volume of the 3D region can therefore be estimated by the summation of *V*_*k*_ accordance with which *k* is the number of delimited regions that compose the 3D region.

### ABC/2 Method

The ABC/2 method is, to date, the conventional approach for calculating the volume of cerebral hematoma with the assumption that A, B, and C are the axes of an ellipsoid. Typically, radiologists do the calculation of these axes. When π is approximated as 3, the ellipsoid’s volume can be determined by

$$V=\frac{4}{3}\pi (\frac{A}{2})(\frac{B}{2})(\frac{C}{2})=\frac{ABC}{2}.$$

If a 3D region appears in the image sheets *X*_*i*_,*…*,*X*_*j*_, in practice, the A axis is first chosen by selecting the sheet containing the longest axis. Then, the minor axis B is selected as the perpendicular segment of the first chosen axis. Finally, the axis C is calculated by the product of the number of sheets in which the region appears and the distance *ΔX*.

To standardize the selection of axes, we introduced the PCA approach, which computes those axes automatically. The approach of PCA is described in full in an article by Abdi and Williams [22].

Following the method ABC/2, as described previously, we employed the PCA method for obtaining A and B. Briefly, the information from the shaded region of each image sheet is represented as a binary digit. Therefore, we located the coordinate containing 1 as (*x*, *y*) that corresponds to the grid representing the data in two-dimensional space. The values of *x* and *y* are centered on 0 by subtracting the mean of the *x* and *y* values from each of those values. By seeing this data as a matrix, the covariance matrix of the data is computed. Next, the eigenvalues and corresponding eigenvectors of the covariance matrix were generated. Then, the data was transformed into a new coordinate system which has two rotated perpendicular axes, *x’* and *y’*. The *x’* axis represents the direction of maximum variation through the data while the *y’* axis is added orthogonally to the new x-axis and positioned to represent the next highest variation through the data. On the new coordinate system, the parameter A is obtained by finding the maximum range of *x’* values along the y’-axis. Meanwhile, the parameter B is the maximum range of *y’* values along x’-axis. The parameter C can be selected using the same technique as in the ABC/2 method.

Since the data is represented as voxels, we can also define the three-dimensional coordinate system and locate the point corresponding to the value 1. The PCA in 3D space can be applied using the decomposition of a three-dimensional array to provide three perpendicular axes *x’*, *y’* and *z’* in a similar manner to the PCA in 2D. The parameter A is the range of *x’* where *y’* and *z’* are zero. In addition, the parameter B is the range of *y’* where *x’* and *z’* are zero. In the same way, the parameter C is the range of *z’* where the others are zero.

### Clinical volume assessment by radiologists

From each CT scan we retrieved the volumes of EDH and IPH manually measured by radiologists from clinical reports. Usually, only the largest EDH and IPH regions identified by radiologists were assessed using the ABC/2 method. Using the Synapse Picture Achiving and Communication System, radiologists scanned through all slides of the CT scan and then identified the largest EDH and IPH. The lengths of the A, B and C axes were measured using a ruler tool. Lastly, the volume of the hemorrhage region was calculated.

### Shape analysis

To assess the efficiency of the volume computation, we anticipated that the ABC/2 approach would be precise if there was a tendency for the approximated form to be an ellipsoid. According to Wadell [23] and Cruz-Matas et al. [19], the roundness of a form is determined by the ratio of its average radius of curvature to the diameter of its greatest inscribed circle.

In this study, the proposed method described by Cruz-Matas et al. [19] was used to calculate the roundness index for voxel-representative three-dimensional data. Assuming briefly that a set of voxels in three-dimensional space is available, we utilized the PCA approach to calculate the primary axes, the lengths of axes (a, b, c) sorted in descending order and regarded as the reference ellipsoid. For each border voxel, the distance between the voxel’s position and the central projection of the reference ellipsoid onto the ellipsoid was then calculated. The roundness index R is the difference between the sum of the distance normalized by the geometric mean of the length of the axes and 1. That is to say

$$R=1-\frac{10}{n{\left(abc\right)}^{\frac{1}{3}}}\sum_{i=1}^{n}\Delta_{k}$$

where *n* is the number of border voxels and ten is the weight multiplied in computation. If *R* approaches 1, this index indicates that the form tends to be an ellipsoid.

### Statistical analysis

The estimation performances of each investigating method are reported as relative error. The relative error can be computed by

$$Relative\;Error=\frac{\left|Exact\;volume-Estimated\;volume\right|}{Exact\;volume.}$$

In the case of phantom data, the relative error could be directly calculated as the exact volume of water filling each of the phantoms was known. Since the exact volumes of hemorrhage in the real dataset are unknown, we utilized the planimetry method as a benchmark for comparing other methods due to the straightforward computation from the input data and the comparison with phantom experiments with the reliable error bounds. Thus, the relative error was calculated, for the real dataset, by

$$Relative\;Error=\frac{\left|Volume\;estimated\;by\;planimetry\;method-Volume\;estimated\;by\;comparing\;method\right|}{Volume\;estimated\;by\;planimetry\;method}.$$

To analyze the agreement between the planimetry method and the others, a Bland–Altman plot [24] was used. We used the plots to visualize the differences in volume measurements between the planimetry method against the truncated pyramidal method, the ABC/2 method through PCA in 2D and PCA in 3D methods. The Bland–Altman plots display the mean difference between two methods of measurement illustrating bias and 95% confidence interval of limits of agreement. The limits of agreement are defined as the mean difference ±1.96 SD of differences [25]. Each hemorrhage region was a data piece in our analysis. It was separately and individually computed and analyzed although some regions were from the same patient. Except in the comparison between clinically employed and computational methods, the volume of the largest EDH and IPH regions for each patient was analyzed because radiologists identified and measured the volume of only the largest EDH and IPH regions. In addition, the Wilcoxon signed-rank test with a significance level of 0.05 was adopted for the statistical analysis of the location of the volumes estimated by two methods. The statistical tests were performed under the alternative hypothesis that the mean of the volumes estimated by one method is significantly higher than the mean of the volumes estimated by another method. To analyze the relationship between the two variables, Spearman’s rank correlation coefficient was calculated. A p-value of 0.05 being used to indicate statistical significance.

To investigate to the efficacy of implementing the appropriate methods with real data, we determined the relationship between the roundness index and the relative error of volume of each method in comparison to the result of the planimetry method. To interpret these relationships, a linear regression was performed.

We analyzed the agreement of the surgical treatments between nerosurgeon’s judgment and the criterion decided based on the volume of the hemorrhages measured by the radiologists and the planimetry method. For the decision based on the EDH volume, surgery was carried out on the patients if the volume of the largest EDH was greater than or equal to 30 cm^3^; otherwise, the decision was observation. The decision of the need for surgery was agreed upon, with the final decision being if the patient required a craniotomy to remove the EDH. In the case of the IPH, surgery was deemed necessary if the volume of the largest IPH on the frontal and temporal lobe was greater than or equal to 30 cm^3^ or was greater than or equal to 50 cm^3^ for other areas. The decision of the need for surgery was agreed upon with the final surgical decision being if the patient required a craniotomy to remove the IPH. The kappa statistic was adapted in this analysis to measure the interobserver agreement between two raters. Cohen’s kappa coefficients (κ) were calculated. If the two raters are in complete agreement, then the kappa coefficient is equal to 1. The coefficient of 0 means the amount of agreement can be expected from random chance, or there is no agreement among the raters. If the kappa coefficient is negative there is no relationship between the ratings of the two raters, or it may reflect a tendency of the raters to give differing ratings [26].

## Results

### Result from investigations of the phantoms

The experiments were performed with six phantoms using the planimetry method, truncated pyramidal method, the ABC/2 method through PCA in 2D, and the PCA in 3D method. The roundness index was also calculated and shown in Table 1. Table 2 shows the relative error of the four methods with respect to the exact volumes.

**Table 1:** Volume calculations from the four methods: Planimetry method, truncated pyramidal method, ABC/2 method through PCA in 2D and PCA in 3D method.

*Shape*	*Roundness index*	*Exact Volume (cm* ^*3*^ *)*	*Planimetry method (cm* ^*3*^ *)*	*Truncated Pyramidal Method (cm* ^*3*^ *)*	*ABC/2 (PCA in 2D) (cm* ^*3*^ *)*	*PCA in 3D (cm* ^*3*^ *)*
Ellipsoid	0.9452	58.00	61.08	61.03	63.60	67.95
Convex lens	0.9164	27.00	26.48	26.46	32.28	35.45
Rough shape	0.8690	28.00	26.71	26.68	29.32	39.56
Triple pants	0.8663	68.00	68.68	69.04	69.59	93.03
Pair of pants	0.8662	59.00	59.22	59.46	58.92	74.76
Knobby ellipsoid	0.8327	36.00	35.09	35.06	64.60	87.47

**Table 2:** Relative error of four methods with respect to exact volume; planimetry method, truncated pyramidal method, ABC/2 method through PCA in 2D, and PCA in 3D with roundness index.

*Shape*	*Planimetry method*	*Truncated Pyramidal Method*	*ABC/2 (PCA in 2D)*	*PCA in 3D*
Ellipsoid	0.0530	0.0522	0.0965	0.1715
Convex lens	0.0193	0.0198	0.1956	0.3130
Rough shape	0.0461	0.0472	0.0470	0.4130
Triple pants	0.0100	0.0154	0.0234	0.3681
Pair of pants	0.0037	0.0079	0.0013	0.2671
Knobby Ellipsoid	0.0253	0.0262	0.7944	1.4297

We determined from Table 1 that the recovered voxel count from the phantom of the ellipsoid is the most similar to the ellipsoid given by the roundness index, followed by the convex lens shape and the rough shape. Table 2 shows that the relative errors of the approximate volumes computed by the planimetry method are the lowest of the other methods, while PCA in 3D gives the highest error. According to our hypothesis, the PCA in 3D will be more accurately approximated when the shape approaches an ellipse. Although the table indicates that the relative error of the PCA in the 3D method is greater than the ABC/2 method through PCA in 2D, it can be explained by the PCA in 3D used in our experiment involved the use of real data to determine the axes, whereas PCA in 2D used the z-axis derived from the number of slides, which is the imaginary axis.

### Results with the real data set

In the case of EDH, the median volumes (Interquartile range: IQR) measured by planimetry, truncated pyramidal, ABC/2 (PCA in 2D) and PCA in 3D methods were 9.23 (41.59), 9.21 (41.56), 10.28 (57.68) and 18.27 (123.86) cm^3^, respectively. Meanwhile, the median volumes (IQR) of IPH measured by planimetry, truncated pyramidal, ABC/2 (PCA in 2D) and PCA in 3D methods were 1.60 (5.51), 1.58 (5.49), 1.48 (8.04) and 2.78 (11.24) cm^3^, respectively.

**Fig 4:**
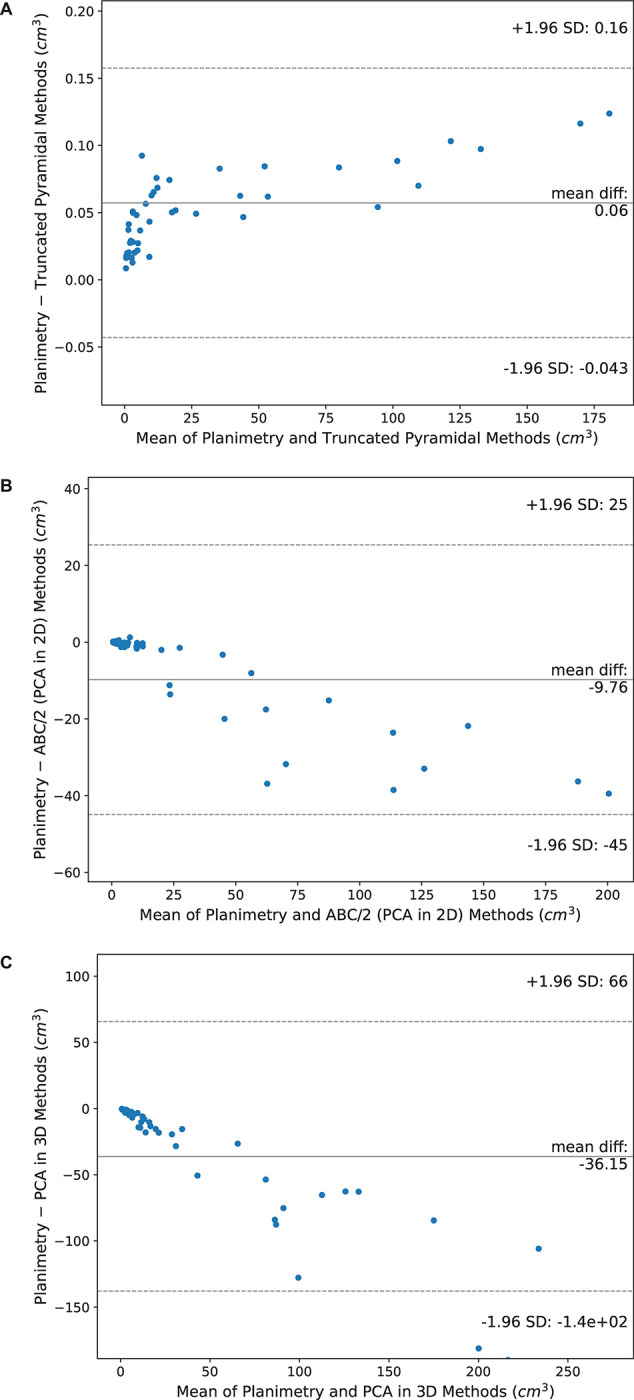
Bland–Altman plots of planimetry against three volume assessment methods in measuring the volume of EDH. (A) truncated pyramidal, (B) ABC/2 method through PCA in 2D and (C) PCA in 3D methods.

As shown in Fig 4, the Bland–Altman plots of planimetry against truncated pyramidal, ABC/2 method through PCA in 2D and PCA in 3D methods in measuring the volume of EDH, show that the volumes estimated by the truncated pyramidal method were slightly lower than those computed by the planimetry method (p<0.001) with a mean (a limit of agreement) of 0.06 (between 0.16 and –0.04) cm^3^. Meanwhile, the ABC/2 method through PCA in 2D and PCA in 3D tended to estimate the volumes of EDH higher than the volumes calculated by the planimetry method (p<0.001) with a mean (a limit of agreement) of –12.67 (between 32.52 and –57.85) cm^3^ and a mean (a limit of agreement) of –28.37 (between 58.70 and –115.51) cm^3^, respectively.

**Fig 5:**
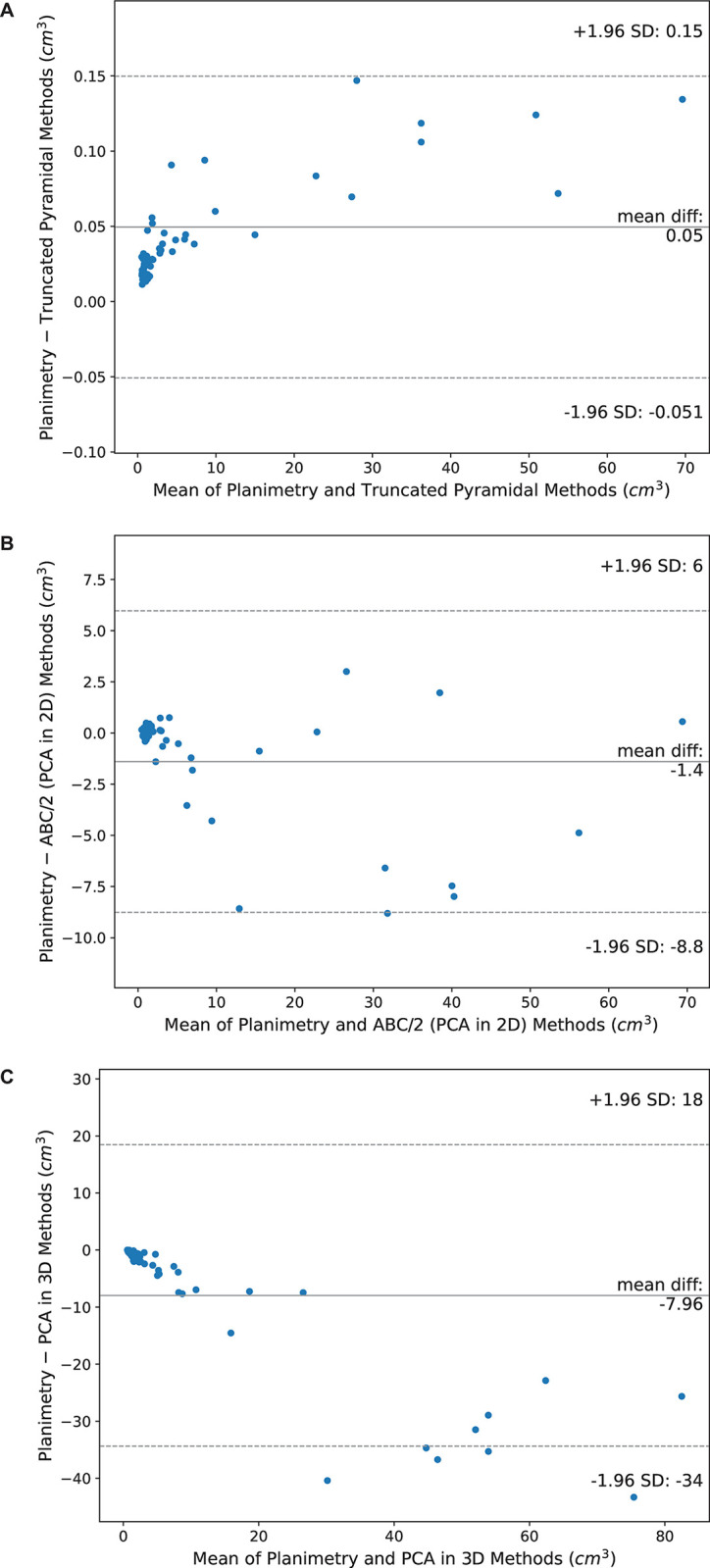
Bland–Altman plots of planimetry against three volume assessment methods in measuring the volume of IPH. (A) truncated pyramidal, (B) ABC/2 method through PCA in 2D and (C) PCA in 3D methods.

The Bland–Altman plots of planimetry for estimating IPH volumes are shown in Fig 5. As can be seen from the figure, the truncated pyramidal method estimated the volumes of IPH as considerably lower than the volumes calculated using the planimetry method (p<0.001). The mean difference between the planimetry and truncated pyramidal methods (a limit of agreement) was 0.05 (between 0.15 and –0.05) cm^3^. In contrast, the volumes computed by the ABC/2 method through PCA in 2D were higher than those calculated by the planimetry method (p<0.001) with a mean difference (a limit of agreement) of –2.07 (between 8.58 and –12.72) cm^3^. In addition, calculations from the PCA in 3D resulted in considerably higher volumes than the planimetry method (p<0.001) with a mean difference (a limit of agreement) of –4.28 (between 9.56 and –18.11) cm^3^. Noticeably, the mean differences in the planimetry against ABC/2 method through PCA in 2D and PCA in 3D tended to be ***out*** of the limit of agreement when the mean volumes of two methods were high.

### Comparison between roundness and relative error

**Fig 6:**
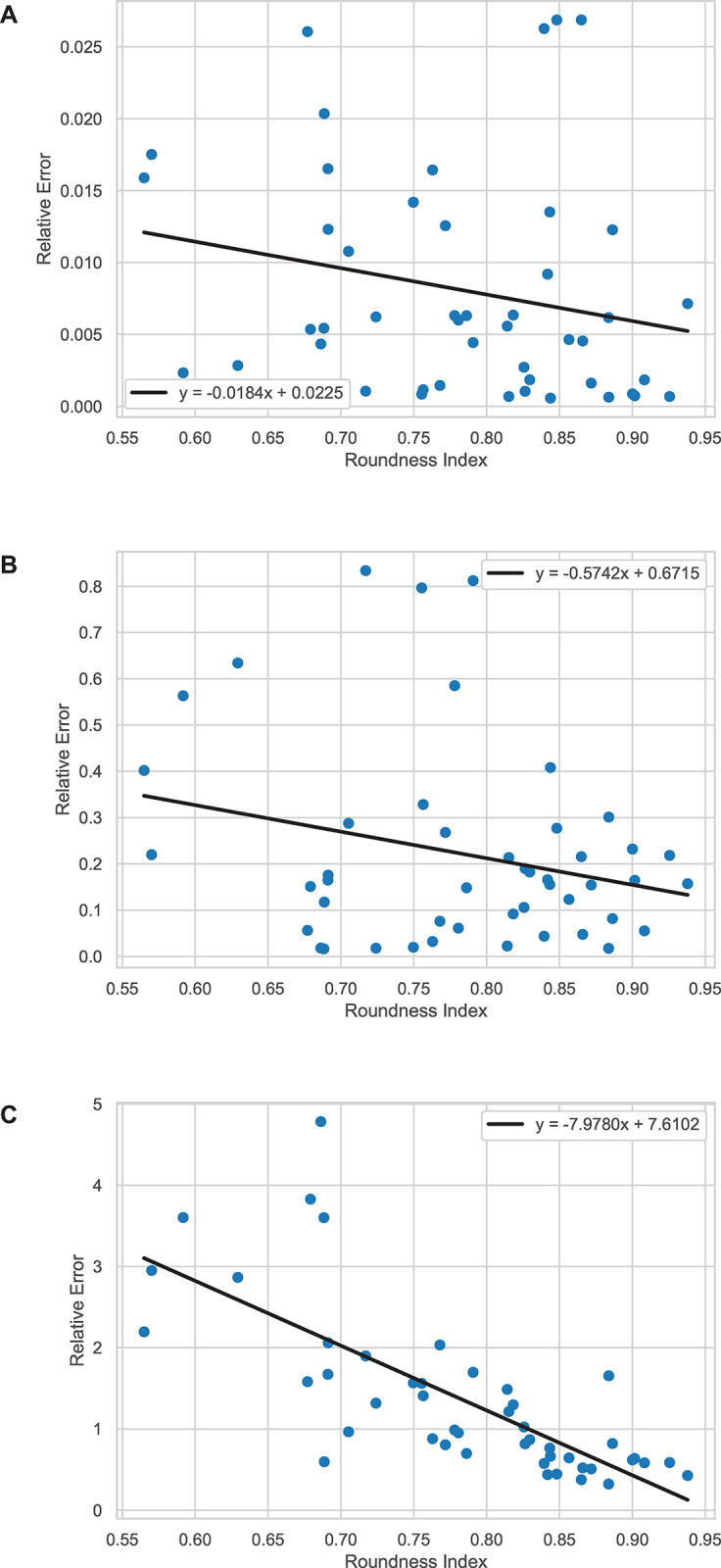
The plots of data between roundness (x-axis) and relative error (y-axis) of each method with respect to the planimetry method for EDH. (A) Truncated pyramidal (B) ABC/2 method through PCA in 2D and (C) PCA in 3D methods.

**Fig 7:**
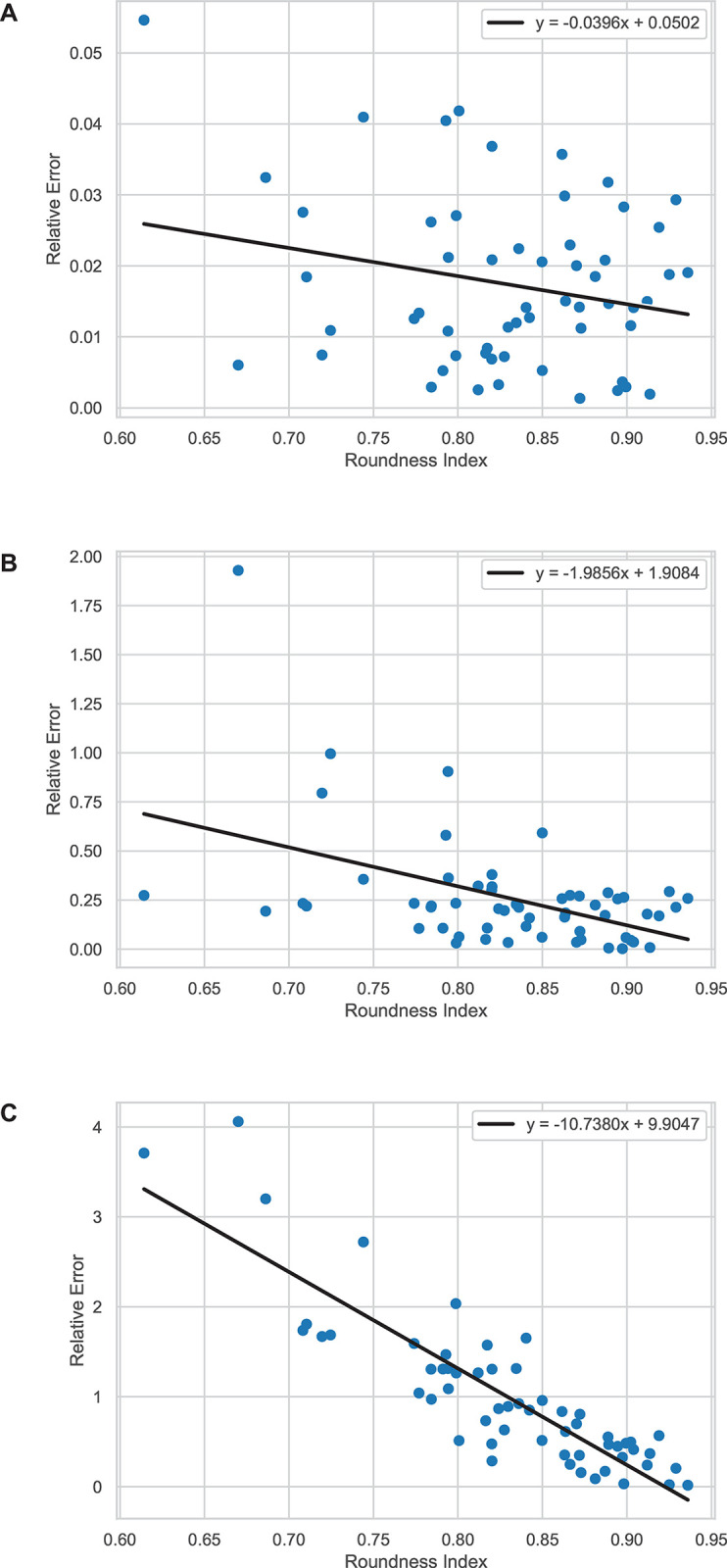
The plots of data between roundness (x-axis) and relative error (y-axis) of each method with respect to the planimetry method for IPH. (A) Truncated pyramidal (B) ABC/2 method through PCA in 2D and (C) PCA in 3D methods.

Figs 6 and 7 illustrate the relationships between roundness index and relative error of volume for each method. In the case of the data pertaining to EDH, there is no relationship between the relative error and roundness index for the truncated pyramidal method with a Spearman’s rank correlation coefficient of –0.285 (p = 0.052). In addition, the relative errors provided by the ABC/2 method through PCA in 2D did not show any statistically significant correlation to the roundness index with a correlation coefficient of –0.091 (p = 0.540). In addition, the relative errors calculated from the data from the PCA in 3D method showed a statistically monotonic decrease against roundness index with a correlation coefficient of -0.795 (p<0.001). However, in the case of IPH, both the ABC/2 through PCA in 2D and PCA in 3D methods showed a negative relationship between their relative error and roundness index with correlation coefficients of –0.366 (p = 0.002) and –0.851 (p<0.001), respectively. The relative errors from the ABC/2 and the truncated pyramidal method did not show any relationship to the roundness index with a correlation coefficient of –0.063 (p = 0.637).

### Comparison between clinically employed and computational assessments

**Fig 8:**
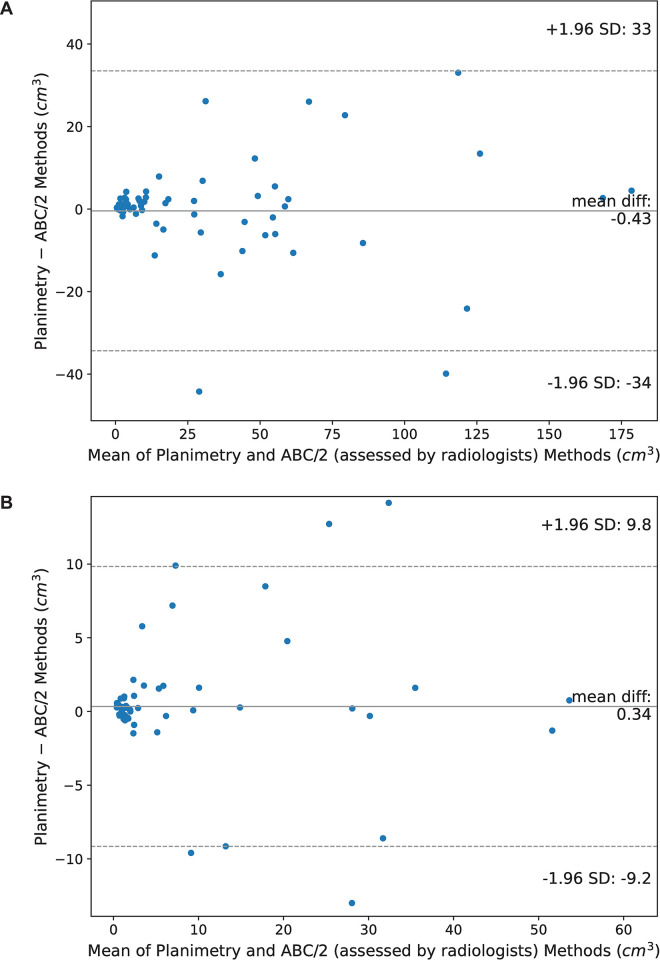
Bland–Altman plots of planimetry method against radiologists in measuring the volume of (A) EDH and (B) IPH.

The Bland–Altman plots of the planimetry method against radiologists in measuring the volume of EDH and IPH are shown in Fig 8. The volumes of EDH assessed by the radiologists were slightly higher than those estimated by the planimetry method (p = 0.077) with a mean (a limit of agreement) of -0.43 (between 33 and –34) cm^3^. In contrast, the volumes estimated by the radiologists were insignificantly lower than those calculated by the planimetry method (p = 0.941) with a mean difference (a limit of agreement) of 0.34 (between 9.8 and –9.2) cm^3^. Interestingly, the mean differences in the planimetry method against radiologists were out of the limit of agreement for both EDH and IPH when the mean volumes of the two assessments were high.

### Effects on surgical treatment

**Fig 9:**
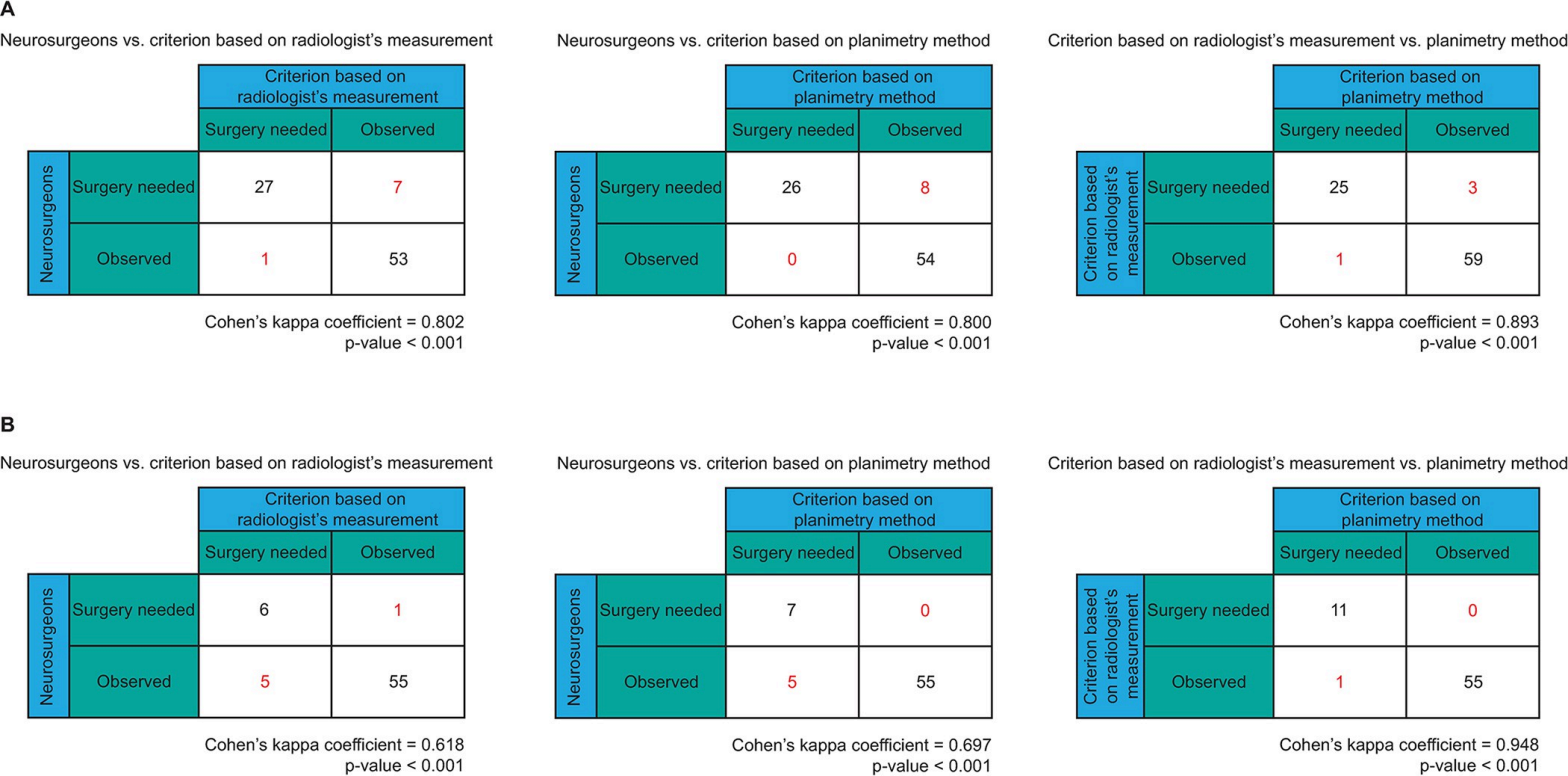
The agreements with Cohen’s Kappa statistics among the surgical treatments decided based on: (A) EDH and (B) IPH volumes measured by the radiologists and the planimetry method and the treatments decided by neurosurgeons.

Fig 9 shows the Cohen’s kappa statistics of agreement among the surgical treatments decided based on the hemorrhage volumes measured by the planimetry method and radiologists and the treatments decided by neurosurgeons. In the case of EDH, the results demonstrated substantial agreement between the judgement of the neurosurgeons versus the criterion based on the EDH volumes measured by the planimetry method and the radiologist with a kappa coefficient of 0.800 and 0.802, respectively. The different decisions made by neurosurgeons and determined by the planimetry method were presented to eight patients. Also, in eight patients the neurosurgeons made different decisions from the treatments recommended by radiologists. We also found data pertinent to four patients in which the surgical treatments decided on the volumes assessed by radiologists differed from those estimated by the planimetry method. The agreement between the radiologists and the planimetry method was almost perfect with a kappa coefficient of 0.893. Considering surgical decisions based on IPH, the agreement between the neurosurgeons versus IPH volumes measured by the planimetry method was substantial with a kappa coefficient of 0.697. In five patients the neurosurgeons disagreed with surgical treatments indicated by the planimetry method. However, there was a substantial agreement between neurosurgeons and IPH volumes measured by radiologists when the kappa coefficient was 0.618. The different decisions were made on six patients. Additionally, the surgical treatments recommended by radiologists using the planimetry method differed in only one patient. The agreement between radiologists and the planimetry method was almost perfect with a kappa coefficient of 0.948.

In addition, we inspected cases in which there were disagreements between methods. In six of the ten cases of patients with EDH where there was a discrepancy between the neurosurgeon’s judgment and the criterion based on the volumes determined by the radiologist and planimetry method, the volume determined by both techniques was less than 30 cm^3^. However, these patients met the requirements for surgery because the EDH thickness was more than 1.5 cm. A case of EDH with a depressed skull fracture, which led to an overestimation of the volume of EDH by the radiologist, was one of the two instances where there was disagreement between the judgement of the neurosurgeon and the volume measured by the radiologist. In another instance, the measurement of the volume by the was underestimated. In two cases, the neurosurgeon’s judgment and the guidelines defined by the volume determined by planimetry did not agree. The planimetry method underestimated the volume of EDH when compared to the radiologist’s measure. The neurosurgeon’s judgment and the criterion from the volume assessed by both methods disagreed in five IPH patients because their volume was compatible with the requirements for surgery yet their GCS scored higher than 8. There was one instance where the neurosurgeon disagreed with the judgment made by the radiologist because the volume measured by the radiologist was underestimated when compared to the planimetry approach.

## Discussions

Compared to the planimetry method, the ABC/2 method through PCA in 2D and PCA in 3D significantly overestimated the volume of both EDH and IPH. However, the truncated pyramidal method resulted in significantly underestimation of the volume in the case of both EDH and IPH. In addition, there was a significant correlation between the roundness index and the relative error in PCA in 2D for IPH and PCA in 3D for both EDH and IPH volumes, indicating that shape influences the calculated volume.

According to the Bland-Altman diagrams, the ABC/2 method through PCA in 2D and PCA in 3D approaches significance regarding overestimation of the volume of both EDH and IPH in comparison to the planimetry method (p<0.001). However, the limits of agreement between these two methods compared to the planimetry method are still large, especially in the case of the PCA in 3D method, which yielded the largest deviation and range of limits of agreement. Therefore, these two methods cannot be used interchangeably with the planimetry method. Previous studies have shown that volume could be either overestimated or underestimated when using ABC/2 in comparison to the planimetry method. Kothari et al. [11] found corresponding levels of overestimation between ABC/2 and planimetric methods. In addition, Scherer et al. [27] reported an overestimation of IPH volume by up to 30% using the ABC/2 method. On the contrary, underestimation in relation to the ABC/2 method was demonstrated in studies by Freeman et al. [9] and Maeda et al. [28]. It should be noted that inaccurate estimation of intracranial hematoma volume using the ABC/2 method influences treatment decision. For example, patients with overestimation of hematoma volume would be selected for surgical rather than conservative treatment and vice versa. Many studies have demonstrated the impact of inaccurate volume estimation using the ABC/2 method. The study by Webb et al. [29] found that the ABC/2 method had an inaccuracy of 2.1% in classifying hematomas with volumes less than 30 ml, when compared to computed tomography-based planimetry, resulting in the exclusion of patients from the study. Moreover, Leary et al. [3] concluded that the computerized measurement for extra-axial hematoma had better predictive power for functional outcome and mortality than the ABC/2 method. On consideration of the truncated pyramidal approach, this procedure statistically significantly underestimated the volume of both EDH and IPH in comparison to the planimetry method (p<0.001). The approach utilizes the connecting of multiple points along the edge of the hematoma to form the lateral surface of the cumulative shape. As suggested by Sun and Sun [16], the limitation of the truncated pyramidal method occurs when there are multiple regions in the same layer as some voxels in the hematoma may not be included in the volume estimation. Therefore, the volume calculated by the truncated pyramidal method is frequently smaller than that calculated using the planimetry method. Please note that the ABC/2 method through computerized PCA in 2D while other studies used manual calculations from the ABC/2 method. The PCA in 2D method may result in an inaccurate estimation of volume by calculating the total volume rather than the individual lesions in the case of two EDHs in close proximity. Consequently, the determination of EDH volume may be inaccurate. However, the use of the ABC/2 method through computerized PCA in 2D decreases the workload of radiologists, increasing estimation accuracy, and the provision of consistent results.

There is a significant monotonic decreasing relationship between the roundness index and relative error for volume estimation of IPH and EDH by PCA in 3D method and IPH by PCA in 2D method in comparison with the planimetry method. EDH volume calculated by PCA in 2D method has a non-monotonic relationship between the roundness index and relative error because the shape of EDH usually has a tendency towards ellipsoidal. Calculation from the PCA in 2D utilizes a similar approach to the ellipsoidal volume formula. It can be implied that IPH volume estimation by the ABC/2 method has a tendency to be more inaccurate than estimation of EDH volume since IPH shape is more rounded. It is remarked that there are some examples of IPH or EDH which have large errors of computing through PCA in 3D when a shape differs from an ellipse, especially in the case of a non-convex shape. The possible reason for the error is that the volume of the approximated ellipsoid is calculated from the non-convex shape.

On the other hand, there is no relationship between the relative error and roundness index in the truncated pyramidal method for both EDH and IPH. The truncated pyramidal method relies on the rectangular subdivision of hematoma and the summation of these meshes. Therefore, using the method that is independent of shape to approximate volume is an efficient method with a lower rate of potential error.

In this era, the use of state-of-art technologies, such as artificial intelligence [3], can be used efficiently to assist physicians in their decision-making by providing high computational power and accessibility [30]. Its use can also decrease the time for assessment in emergency situations. The integration of the findings from many studies [13–15] has shown promising efficient tools for hematoma segmentation. As demonstrated in our investigation and analysis, the planimetry method is an efficient measure of hemorrhage volumes. Although the agreements of surgical decisions based on the volumes of the hemorrhage measured by the planimetry method and radiologists have almost been perfected, the planimetry method was faster and more convenient. In addition, for IPH, the planimetry method provided a higher kappa coefficient of agreement with the neurosurgeon’s judgment. Furthermore, the method facilitated the moderation of agreement of surgical decisions with neurosurgeons which supersedes those made by radiologists. Thus, it is a better choice compared to the ABC/2 method performed by radiologists in calculating hemorrhage volumes.

Integrating the planimetry method with automatic segmentation would be beneficial by providing fast, convenient, and accurate calculation of volume. In addition, these tools may be applied in other situations, such as the calculation of the volume of brain tumors to monitor response after treatment. Going forward, the co-involvement of an effective computerization system with the expertise of radiologists may well be the best approach.

## Conclusions

The estimation error of hematoma volume was associated using the ABC/2 method, especially in the case of IPH. Comparing four volume estimation techniques, the planimetry method has the lowest level of error with regard to volume calculation and is also independent of the shape of the hematoma. Furthermore, the experiments show an acceptable agreement of surgical treatments between the neurosurgeons versus hemorrhage volumes measured by the planimetry method. Thus, our study highlights the potential of the planimetry method applied with automatic segmentation for the evaluation of EDH and IPH volume with increased accuracy and convenience, allowing faster calculation with consistent results.
